# Effects of external neuromuscular electrical stimulation in women with urgency urinary incontinence: a randomized sham-controlled study

**DOI:** 10.1007/s00345-024-05126-7

**Published:** 2024-07-21

**Authors:** Tugba Birben Kurt, Bulent Yilmaz, Seyda Toprak Celenay

**Affiliations:** 1 Department of Neurological Physiotherapy Rehabilitation, Guneysu Vocational School of Physical Therapy and Rehabilitation, Recep Tayyip Erdogan University, Rize, Turkey; 2 The Faculty of Medicine, Department of Gynecology and Obstetrics, Recep Tayyip Erdogan University, Rize, Turkey; 3 Health Sciences Faculty, Department of Physiotherapy and Rehabilitation, Ankara Yildirim Beyazit University Ankara, Ankara, Turkey

**Keywords:** Urinary incontinence, Urgency, Electrical stimulation therapy, Physiotherapy, Quality of life, Sexual dysfunction

## Abstract

**Background and Purpose:**

The present study aims to investigate the effects of external neuromuscular electrical stimulation (NMES) on urinary symptoms, pelvic floor muscle strength (PFMS), quality of life (QoL), sexual function, perception of subjective improvement (PSI), and satisfaction in urgency urinary incontinence (UUI).

**Materials and Methods:**

The randomized sham-controlled study design was employed in this study. Women aged 18–65 years, who were diagnosed with UUI, were randomly allocated into the NMES (external NMES + lifestyle advice, n = 15) and sham groups (sham NMES + lifestyle advice, n = 15). Both groups performed the application for 30 min, three days a week for eight weeks. Urinary symptoms were evaluated by using the International Incontinence Consultation Questionnaire-Short Form (ICIQ-SF) and a 3-day bladder diary. PFMS was assessed using the Modified Oxford Scale (MOS), QoL using the King’s Health Questionnaire (KHQ), and sexual function using the Pelvic Organ Prolapse/Urinary Incontinence Sexual Function Questionnaire (PISQ-12). The PSI and satisfaction were questioned.

**Results:**

There was a higher level of decrease in the ICIQ-SF score, the mean number of voids/night and UI, all scores related to the KHQ (excluding interpersonal relationships), and a higher level of increase in maximum voiding volume, MOS scores, PISQ-12-emotional, PISQ-12-physical, and PISQ-12-total scores in the NMES group when compared to the sham group (p < 0.05). PSI and satisfaction were at higher levels in the NMES group than in the sham group (p < 0.05).

**Conclusions:**

External NMES was an effective and complementary method in reducing urinary symptoms and improving PFMS, QoL, sexual function, PSI, and satisfaction level in women with UUI.

**Clinical Trial Registration:**

NCT04727983.

## Introduction

Urgency urinary incontinence (UUI), which is the involuntary loss of urine associated with urgency, constitutes 22% of urinary incontinence (UI) in women [1]. The etiopathogenesis of UUI generally includes detrusor overactivity, low detrusor compliance, urothelial sensitivity, and bladder hypersensitivity [1]. It negatively affects the quality of life (QoL) [2].

Conservative treatments, including lifestyle advice, bladder training, pelvic floor muscle exercise (PFME), and electrical stimulation (ES), can be used [1]. Lifestyle advice, such as reducing bladder irritants, weight control, and increasing physical activity, is considered a method for treating UUI according to guidelines [3, 4].

Neuromuscular electrical stimulation (NMES) can improve sensorial awareness, muscle reeducation, and circulation and can be applied transcutaneously, percutaneously, or as an implant [5]. Transcutaneous applications are divided into internal (IES) and external ES (EES). However, external NMES devices, known as novel EES, are preferred more than IES due to disadvantages such as expensive probes, risk of infection, and patient reluctance towards intravaginal-anal application. The number of studies examining the effects of NMES on overactive bladder (OAB) accompanied by UUI is limited [6]. More high-quality studies examining the effects of external NMES on patients with UUI are needed.

Therefore, the present study aims to examine the effects of external NMES on urinary symptoms, pelvic floor muscle strength (PFMS), QoL, sexual function, perception of subjective improvement (PSI), and satisfaction in women with UUI.

## Methods

### Study design

This study, which was planned as a randomized sham-controlled trial, was approved by the clinical research ethics committee of Recep Tayyip Erdogan University (Approval number:2020/07). The Declaration of Helsinki was followed in the research process. The present study, which was carried out at the gynecology and obstetrics outpatient clinic of training and research hospital of the university, was registered at https://www.clinicaltrials.gov (NCT04727983).

### Participants

Volunteer women aged 18–65 years, who were newly diagnosed with UUI or discontinued their drug use for UUI at least one month ago, were included in the present study. Exclusion criteria were pregnancy, urinary infection, advanced pelvic organ prolapse (stage 3 and above [7]), stress urinary incontinence (SUI) (positive cough stress test), mixed urinary incontinence, neurological disease (i.e. Parkinson’s Disease, multiple sclerosis, etc.), loss of sensation involving pelvic region and lower extremity, cardiac arrhythmia, using electronic/metal implants or pacemaker and malignant disease**.** Written consent forms were obtained.

### Results

Physical and demographic characteristics (age and body mass index (BMI)) and clinic histories (number of pregnancies, number of deliveries, constipation) were recorded. Urinary infection was screened using urine analysis at the time of enrollment. No infection was detected in any patient during treatment. Sensorial evaluations were conducted around the thigh, where ES application would be performed. There was no patient describing neurological symptoms or sensational loss. All assessments were conducted by the same therapist before treatment (BT), mid-term (MT, 4th week), and after treatment (AT, 8th week). AT, PSI, and satisfaction level of patients were also questioned.

The urinary incontinence severity was evaluated as the primary outcome by using the International Consultation on Incontinence Questionnaire-Urinary Incontinence Short Form (ICIQ-SF) [8]. Moreover, the participants completed a 3-day bladder diary. The mean number of voids/day, voids/night, UI, and maximum voiding volume were recorded for the urinary symptoms. The PFMS was assessed using the Modified Oxford Scale (MOS) while the woman was in the lithotomy position [9]. The QoL was examined by using the first part of the King’s Health Questionnaire (KHQ), including 9 sub-dimensions [10]. The sexual function was addressed by using the Pelvic Organ Prolapse/Urinary Incontinence Sexual Function Scale (PISQ-12) [11]. The PSI of patients was evaluated through a 4-item Likert-type scale (worse, same, better, cured) [12]. Patient satisfaction was questioned by using a Visual Analog Scale (VAS) consisting of a horizontal line of 10 cm in length, where “0” defines “I am not at all satisfied with the treatment” and “10” defines “very satisfied with the treatment”.

### Randomization

Participants were assigned to one of NMES and sham groups by using a computer-based block randomization list, which was created by an individual not involved in the assessment and the treatment.

### Intervention

External NMES and lifestyle advice were given to the NMES group, whereas sham NMES and lifestyle advice were given to the sham group. Patients received advice on fluid intake, diet, bladder irritants, constipation, and weight control by using a brochure. Compliance with these lifestyle advices was assessed using a 4-item Likert-type scale. None of the patients used medication during the study.

**Fig. 1:**
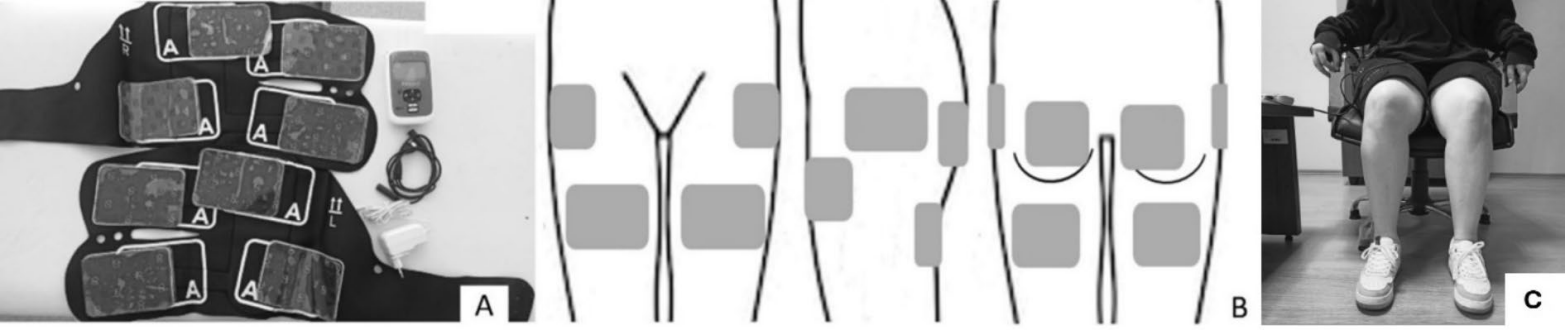
**A** INNOVO® device, **B** positions of electrodes, **C** application of the device

An INNOVO® device (Atlantic Therapeutics, Galway, Ireland) consisting of 2 wearable sleeves and 8 external electrodes was used in NMES application (Fig. 1). The procedure was conducted in a back-supported sitting position with a specific waveform and parameters (10 Hz frequency, 250 µs pulse width, 0.5-s ramp up and down ramp time, 5-s contraction, and 0-s relaxation periods). In the Sham NMES, the electrodes were connected as in the NMES group and the light and the sound of the device were turned on, but the current was not increased. Patients were blinded to the intervention modalities administered to the groups. Both groups received the interventions for 30 min, three days a week for eight weeks.

### Sample size and statistical analysis

G*Power (v.3.9.1.7) program was used to determine the sample size. A pilot study carried out on 10 women revealed effect sizes for ICIQ-SF, MOS, KHQ-incontinence effect, and PISQ-total scores as 0.675, 0.670, 0.519, and 0.557, respectively. At least 14 patients per group were needed at 90% power (α = 0.05, β = 0.10). An additional 20% of patients were added to account for data losses, resulting in a total of at least 34 patients for the study.

The normal distribution of variables was determined using the Shapiro–Wilk test. Mixed ANOVA was used to analyze the effects of the group factor (NMES and sham group), time factor (BT, MT, and AT), and their interaction. Pairwise comparisons were conducted for the time factor by using the Bonferroni method. The Chi-Square test was used for categorical variables. Statistical analyses were conducted by using IBM SPSS Statistics 21.0, and the statistical significance was set at p < 0.05.

## Results

Forty-four patients were evaluated for eligibility, and 10 patients were excluded for various reasons (advanced pelvic organ prolapse (n = 5), urinary infection (n = 4), urinary retention (n = 1)). After randomization, 1 patient from the NMES group and 2 patients from the sham group withdrew due to COVID-19, and 1 patient from the NMES group withdrew due to relocation. Fifteen patients in the NMES group (age = 47.66 ± 9.59 years, BMI = 28.32 ± 5.93 kg/m^2^, number of pregnancies = 4, number of deliveries = 3, constipation = 8) and 15 patients in the sham group (age = 48.06 ± 9.85 years, BMI = 32.82 ± 6.67 kg/m^2^, number of pregnancies = 3, number of deliveries = 3, constipation = 7) completed the study. Physical, demographic, and clinical characteristics, urinary symptoms, PFMS, QoL, sexual function, PSI, and satisfaction results were similar between groups at baseline (p > 0.05). No adverse effects were reported during the applications.

### Urinary symptoms

A reduction was found in the ICIQ-SF scores between BT-MT, BT-AT, and MT-AT in the NMES group (p < 0.05), while there was a decrease in the ICIQ-SF between BT-AT in the sham group (p < 0.05). There was a higher level of decrease in the ICIQ-SF scores in the NMES group when compared to the sham group (for group*time interaction (GTI) p < 0.05, Table 1).

**Table 1:** Comparison of urinary symptoms, of MOS and of PISQ-12 values according to groups and time

	NMES group (*n* = 15)		Sham group (*n* = 15)		*P* (BG)	*P* (GTI)
	X ± SD	Median (IQR)	X ± SD	Median (IQR)		
*ICIQ-SF Total*						
BT	15.80 ± 2.95	16.00 (5.00)	16.67 ± 1.91	17.00 (2.00)	< 0.001*	< 0.001*
MT	10.60 ± 3.98	11.00 (4.00)	15.33 ± 3.52	16.00 (5.00)		
AT	6.13 ± 4.75	6.00 (6.00)	14.60 ± 3.11	15.00 (4.00)		
*P* (Time)	< 0.001*		0.020*			
*P* (WG)						
BT- MT	< 0.001*		0.114			
BT-AT	< 0.001*		0.005*			
MT -AT	< 0.001*		0.085			
*Mean number of voids/day*						
BT	7.68 ± 3.31	7.00 (4.00)	7.14 ± 3.35	6.70 (3.00)	0.904	0.544
MT	6.48 ± 1.93	6.00 (2.30)	7.13 ± 3.62	6.30 (2.50)		
AT	5.93 ± 1.77	6.00 (2.00)	6.68 ± 3.32	6.00 (3.40)		
*P* (Time)	0.013*		0.585			
*P* (WG)						
BT- MT	0.033*					
BT-AT	0.015*					
MT -AT	0.139					
*Mean number of voids/night*						
BT	2.05 ± 3.72	1.00 (1.67)	1.23 ± 0.91	1.30 (1.70)	0.216	0.033*
MT	1.00 ± 1.27	1.00 (1.30)	1.25 ± 0.99	1.30 (2.00)		
AT	0.57 ± 0.90	0.00 (1.00)	1.21 ± 0.96	1.00 (1.70)		
*P* (Time)	< 0.001*		0.939			
*P* (WG)						
BT- MT	0.249					
BT-AT	0.004*					
MT -AT	0.432					
*Mean number of urinary incontinences*						
BT	5.96 ± 10.48	3.30 (3.70)	2.13 ± 2.31	1.60 (2.70)	0.881	< 0.001*
MT	2.59 ± 5.11	0.60 (2.80)	1.76 ± 2.03	1.30 (2.20)		
AT	1.33 ± 2.80	0.30 (2.00)	1.63 ± 1.72	1.00 (1.00)		
*P* (Time)	0.037*		0.024*			
*P* (WG)						
BT- MT	0.036*		0.041*			
BT-AT	0.038*		0.022*			
MT -AT	0.077		0.443			
*Maximum voiding volume (ml)*						
BT	392.67 ± 154.71	440.00 (270.00)	471.67 ± 174.96	500.00 (300.00)	0.494	0.013*
MT	409.67 ± 145.03	450.00 (250.00)	445.67 ± 164.67	500.00 (345.00)		
AT	443.67 ± 154.32	500.00 (250.00)	446.33 ± 159.98	500.00 (300.00)		
*P* (Time)	0.010*		0.247			
*P* (WG)						
BT- MT	0.252					
BT- AT	0.018*					
MT- AT	0.022*					
*MOS values*						
BT	1.07 ± 1.03	1.00 (1.00)	0.73 ± 0.88	1.00 (1.00)	0.003*	< 0.001*
MT	1.93 ± 1.03	2.00 (1.00)	0.80 ± 0.86	1.00 (1.00)		
AT	2.53 ± 0.91	2.00 (1.00)	0.80 ± 0.86	1.00 (1.00)		
*P* (Time)	< 0.001*		0.334			
*P* (WG)						
BT- MT	0.004*					
BT-AT	< 0.001*					
MT -AT	0.001*					
*PISQ-12 emotional*						
BT	5.00 ± 4.08	6.00 (7.00)	4.13 ± 3.62	5.00 (6.00)	0.071	0.001*
MT	7.07 ± 4.15	8.00 (7.00)	4.40 ± 3.48	4.00 (7.00)		
AT	7.87 ± 4.25	10.00 (7.00)	4.00 ± 3.58	4.00 (6.00)		
*P* (Time)	0.005*		0.285			
*P* (WG)						
BT- MT	0.019*					
BT-AT	0.004*					
MT -AT	0.034*					
*PISQ-12 physical*						
BT	10.87 ± 6.71	14.00 (12.00)	10.07 ± 7.25	13.00 (16.00)	0.336	0.013*
MT	11.53 ± 6.45	11.00 (10.00)	9.73 ± 6.49	10.00 (11.00)		
AT	12.67 ± 6.58	15.00 (8.00)	9.40 ± 6.71	11.00 (16.00)		
*P* (Time)	0.037*		0.441			
*P* (WG)						
BT- MT	**0.329**					
BT-AT	0.041*					
MT -AT	0.045*					
*PISQ-12 partner dependent*						
BT	5.27 ± 3.93	6.00 (7.00)	6.13 ± 4.37	6.00 (9.00)	0.916	0.354
MT	5.93 ± 3.92	7.00 (6.00)	6.20 ± 4.33	6.00 (8.00)		
AT	6.20 ± 4.03	8.00 (7.00)	5.73 ± 4.58	6.00 (9.00)		
*P* (Time)	0.259		0.732			
*PISQ-12 Total*						
BT	21.13 ± 11.72	24.00 (15.00)	20.93 ± 14.14	24.00 (32.00)	0.315	< 0.001*
MT	24.47 ± 11.38	27.00 (11.00)	20.20 ± 12.14	21.00 (16.00)		
AT	26.67 ± 12.37	31.00 (9.00)	18.93 ± 13.12	23.00 (32.00)		
*P* (Time)	0.001*		0.398			
*P* (WG)						
BT- MT	0.023*					
BT-AT	0.003*					
MT -AT	0.022*					

*P < 0.05, *BT* Before Treatment, *MT* Mid-term(4th week), *AT* After Treatment, *BG* Between group (comparison between groups at same time), *WG* Within group (comparison within group), *GTI* Group*time interaction (comparison of change in groups over time), *NMES* Neuromuscular electrical stimulation, *ICIQ-SF* International Incontinence Consultation Questionnaire-Short Form, *MOS* Modified Oxford Scale, *PISQ-12* Pelvic Organ Prolapse/Urinary Incontinence Sexual Function Questionnaire-12

In the NMES group, there was a decrease in the number of voids per day and night (BT-MT and BT-AT) and the number of urinary incontinences (UI) (BT-MT and BT-AT). Additionally, an increase was observed in maximum voiding volume between BT-AT and MT-AT (p < 0.05). In the sham group, a decrease was found between BT-MT and BT-AT in only the mean number of UI (p < 0.05). A higher level of decrease was observed in the number of voids/night and the number of UI and a higher level of increase in maximum voiding volume in the NMES group when compared to the sham group (for GTI p < 0.05, Table 1).

### PFMS

An increase was found in MOS scores between BT-MT, BT-AT, and MT-AT in the NMES group (p < 0.05). No improvement was found in the sham group (p > 0.05). A higher level of increase was observed in MOS scores in the NMES group when compared to the sham group (for GTI p < 0.05, Table 1).

### QoL

A decrease was determined in KHQ-general health, KHQ-social and KHQ-personal relations limitation scores of KHQ between BT-AT and MT-AT and also in KHQ-incontinence effect, KHQ-physical and KHQ-role limitation scores, KHQ-emotional status, KHQ-sleep-energy disturbance, and KHQ-severity scores between BT-MT, BT-AT, and MT-AT in the NMES group (p < 0.05). No change was observed in the sham group (p > 0.05). There was a higher level of decrease in KHQ scores (excluding interpersonal relationships) in the NMES group than in the sham group (for GTI p < 0.05, Table 2).

**Table 2:** Comparison of of KHQ scores according to groups and time

	NMES group (*n* = 15)		Sham group (*n* = 15)		*P* (BG)	*P* (GTI)
	X ± SD	Median (IQR)	X ± SD	Median (IQR)		
*KHQ-general health*						
BT	51.67 ± 17.59	50.00 (25.00)	55.00 ± 16.90	50.00 (0.00)	0.011*	0.028*
MT	40.00 ± 12.68	50.00 (25.00)	48.33 ± 19.97	50.00 (0.00)		
AT	25.00 ± 18.89	25.00 (50.00)	48.33 ± 14.84	50.00 (0.00)		
*P* (Time)	< 0.001*		0.207			
*P* (WG)						
BT- MT	0.068					
BT-AT	0.001*					
MT -AT	0.003*					
*KHQ -incontinence impact*						
BT	86.67 ± 16.90	100.00 (33.33)	82.22 ± 24.77	100.00 (33.33)	0.040*	< 0.001*
MT	53.33 ± 27.60	66.67 (33.33)	67.78 ± 23.96	66.67 (50.00)		
AT	28.89 ± 30.52	33.33 (33.33)	68.89 ± 15.26	66.67 (0.00)		
*P* (Time)	< 0.001*		0.071			
*P* (WG)						
BT- MT	< 0.001*					
BT-AT	< 0.001*					
MT -AT	< 0.001*					
*KHQ -role limitations*						
BT	66.67 ± 26.73	66.67 (50.00)	62.22 ± 32.41	66.67 (50.00)	0.043*	< 0.001*
MT	47.78 ± 28.78	33.33 (33.33)	62.22 ± 23.96	66.67 (0.00)		
AT	17.78 ± 22.24	0.00 (33.33)	56.67 ± 28.03	66.67 (33.33)		
*P* (Time)	< 0.001*		0.490			
*P* (WG)						
BT- MT	0.138					
BT-AT	0.021*					
MT -AT	0.041*					
*KHQ -physical limitations*						
BT	67.78 ± 23.96	66.67 (33.33)	55.56 ± 37.08	50.00 (83.33)	0.166	< 0.001*
MT	38.89 ± 20.57	33.33 (33.33)	55.56 ± 29.99	66.67 (33.33)		
AT	15.56 ± 17.21	0.00 (33.33)	46.67 ± 30.98	50.00 (50.00)		
*P* (Time)	< 0.001*		0.198			
*P* (WG)						
BT- MT	0.002*					
BT-AT	< 0.001*					
MT -AT	0.001*					
*KHQ -social limitations*						
BT	52.59 ± 28.32	44.44 (44.44)	42.96 ± 34.34	33.33 (55.56)	0.366	< 0.001*
MT	40.74 ± 28.68	22.22 (44.44)	43.70 ± 27.37	44.44 (44.44)		
AT	13.33 ± 19.34	0.00 (22.22)	42.22 ± 25.96	44.44 (44.44)		
*P* (Time)	< 0.001*		0.900			
*P* (WG)						
BT- MT	0.060					
BT-AT	< 0.001*					
MT -AT	0.003*					
*KHQ -personal relationship*						
BT	23.33 ± 37.16	0.00 (50.00)	25.56 ± 36.66	0.00 (50.00)	0.304	0.161
MT	16.67 ± 31.49	0.00 (33.33)	30.00 ± 38.93	0.00 (66.67)		
AT	7.78 ± 18.76	0.00 (0.00)	28.89 ± 39.57	0.00 (66.67)		
*P* (Time)	0.011*		0.395			
*P* (WG)						
BT- MT	0.138					
BT-AT	0.021*					
MT -AT	0.041*					
*KHQ -emotions*						
BT	60.74 ± 36.34	66.67 (77.78)	54.07 ± 33.82	44.44 (55.56)	0.145	< 0.001*
MT	45.93 ± 28.75	33.33 (44.44)	51.11 ± 37.27	66.67 (77.78)		
AT	14.81 ± 19.09	11.11 (22.22)	58.52 ± 30.99	66.67 (44.44)		
*P* (Time)	< 0.001*		0.479			
*P* (WG)						
BT- MT	0.036*					
BT-AT	< 0.001*					
MT -AT	< 0.001*					
*KHQ -sleep/energy*						
BT	48.89 ± 27.07	50.00 (33.33)	44.44 ± 19.58	50.00 (33.33)	0.021*	< 0.001*
MT	34.44 ± 17.21	33.33 (0.00)	49.63 ± 24.71	50.00 (33.33)		
AT	17.78 ± 20.38	0.00 (33.33)	51.11 ± 21.33	50.00 (33.33)		
*P* (Time)	< 0.001*		0.391			
*P* (WG)						
BT- MT	0.004*					
BT-AT	0.001*					
MT -AT	0.003*					
*KHQ-severity measures*						
BT	52.89 ± 22.74	53.33 (46.67)	63.11 ± 24.79	60.00 (26.67)	0.001*	< 0.001*
MT	34.22 ± 17.97	33.33 (33.33)	60.89 ± 20.76	66.66 (26.67)		
AT	16.89 ± 12.31	13.33 (20.00)	56.89 ± 23.07	53.33 (33.33)		
*P* (Time)	< 0.001*		0.145			
*P* (WG)						
BT- MT	0.002*					
BT-AT	< 0.001*					
MT -AT	0.003*					

*P < 0.05, *BT* Before Treatment, *MT* Mid-term(4th week), *AT* After Treatment, *BG* Between group (comparison between groups at same time), *WG* Within group (comparison within a group), *GTI* Group*time interaction (comparison of change in groups over time), *NMES* Neuromuscular electrical stimulation, *KHQ* King’s Health Questionnaire

### Sexual function

An increase was determined in PISQ-12-emotional and PISQ-12-total scores between BT-MT, BT-AT, and MT-AT and in PISQ-12-physical scores between BT-AT and MT-AT in the NMES group (p < 0.05). No improvement was observed in the sham group (p > 0.05). There was a higher level of increase in PISQ-12 scores (excluding partner dependent score) in the NMES group than in the sham group (for GTI p < 0.05, Table 1).

### PSI and satisfaction

PSI results showed that in the NMES group, 60.0% gave the response “I am better” and 40.0% gave the response “I am completely healed”. In the sham group, 53.3% responded by stating “I am the same”, 40.0% by stating “better”, and 6.7% by stating “fully recovered”. There was a significant difference between groups in PSI (p < 0.001), and patient satisfaction was higher in the NMES group (p < 0.001). Regarding compliance with recommendations, 6.7% in the NMES group sometimes applied them, 86.7% applied them mostly, and 6.7% applied them completely. In the sham group, 26.7% sometimes applied them, 66.7% applied them mostly, and 6.7% applied them completely. There was no significant difference between groups in compliance with recommendations (p > 0.05).

## Discussion

The present study revealed that the NMES was effective in reducing urinary symptoms and improving PFMS, QoL, sexual function, PSI, and patient satisfaction levels. There was a higher level of improvement in some urinary symptoms, PFMS, QoL, and sexual function in the NMES group in the early period (MT-4th week) than in the sham group. In the sham group, only urinary symptoms were reduced in the MT and AT.

Generally, low-frequency currents are used in ES applications in the management of UUI [13–15]. Yamanishi et al. [13] divided OAB patients with UUI into ES and sham ES groups. After treatment, UI, urgency, and voiding episodes decreased, and bladder capacity improved in the NMES group more than in the sham group. Franzen et al. [14] compared drug therapy with IES for UUI, finding similar improvements in voiding frequency and bladder capacity. Guo et al. [15] divided stroke patients into two groups and reported that NMES significantly reduced symptoms of UUI in comparison to the sham group. It was found in the present study that NMES decreased incontinence severity and frequency, as well as day and night urination, while increasing maximum voided volume. These results may be because the low-frequency NMES applications stimulating the pudendal nerve afferents could activate the detrusor muscle reflexively, while also reducing detrusor hypersensitivity through sensory awareness and cortical or neural changes [16].

Decreased PFMS may be a risk factor for UUI symptoms [17]. McClurg et al. [18] reported that NMES (40 Hz) with intravaginal probe plus PFME was more effective than sham NMES in reducing incontinence symptoms in UUI patients but had similar effects on PFMS. Celenay et al. [6] stated that external NMES application improved PFMS in women with OAB-wet. In a systematic review, it was reported that NMES could increase PFMS more than PFME [19]. In the present study, PFMS improved in only the NMES group in MT and AT. These results may have originated from low-frequency NMES, increasing blood flow, regeneration, and increasing sensory input [16]. Furthermore, patients with UUI tend to over-contract their pelvic floor muscles to suppress urgency, and thus pelvic floor functions may be impaired [20]. Accordingly, the reduction of UUI symptoms by external NMES may also have contributed to PFMS.

It is known that UUI has more negative effects on QoL than SUI [2]. Studies suggest both IES (5–10 Hz) and drug therapy improve QoL, but drug therapy may have side effects [14, 21]. In the present study, in the NMES group, it was observed that QoL increased in all sub-dimensions in AT, while some sub-dimensions also improved in MT. NMES applied at low frequency can help control urgency reflexively by stimulating pudendal nerve afferents [5, 16]. Thus, UUI can be decreased and QoL may be improved.

Women with UUI often report lubrication and dyspareunia as major complaints with a rate of 34% [22]. Dmochowski et al. [23] reported that both EES and IES had similar effects in reducing SUI and improving sexual function. In the present study, there was an improvement in all parameters related to sexual functions improved more in the NMES group when compared to the sham group, except for partner dependency. Sexual function can be affected by many factors such as physical and psychological factors and the relationship with the partner [21]. These improvements may be due to reducing UUI and improving the sensory awareness of the pelvic floor, blood flow, and vaginal secretion with NMES [24].

A higher level of improvement was found in the NMES group in terms of PSI and patient satisfaction. Furthermore, the PSI values were also high in the sham group. The improvement in sham group is thought to be due to the compliance with lifestyle recommendations, the patient-therapist interaction, and increased patients’ belief in treatment with a device [25].

In conclusion, the external NMES was effective and complementary when reducing urinary symptoms and improving PFMS, QoL, sexual function, PSI, and patient satisfaction in women with UUI. Since the device is affordable and the procedure can be easily conducted at home, it can be recommended to patients as a home program. Long-term follow-up studies examining the effects of external NMES in both men and women with different pelvic floor dysfunctions are needed.

## Data Availability

All data is available in our system. Authors will be contacted for usage.
